# Biosafety protection and workflow of clinical microbiology laboratory under COVID-19: A review

**DOI:** 10.1097/MD.0000000000031740

**Published:** 2022-11-11

**Authors:** Wenjun Zhou, Limin Zou, Fenyong Zhu, Jie Yang

**Affiliations:** a Clinical Laboratory, Danyang People’s Hospital of Jiangsu Province, Danyang Hospital Affiliated to Nantong University, Jiangsu, China.

**Keywords:** biosafety protection, clinical microbiology room, COVID-19, workflow

## Abstract

This paper mainly discusses how to do a good job of daily biosafety protection measures in clinical microbiology laboratories during the epidemic of COVID-19, so as to ensure the safe development of routine clinical microbiology testing items. According to the microbiological and epidemiological characteristics of the novel coronavirus, this paper analyzed the potential risks of the laboratory from the perspective of personal protection before, during, and after testing. Combined with the actual work situation, the improved biosafety protection measures and optimized work flow are introduced to ensure the safety of medical staff and the smooth development of daily work. Danyang People’s Hospital of Jiangsu Province, clinical microbiology laboratory of clinical laboratory in strict accordance with the relevant laws and regulations, technical specifications and the expert consensus, combined with their own conditions, the biosafety measures to perfect the working process was optimized, effectively prevent the laboratory exposure, and maintain strict working condition for a long time, continue to improve. We found that the biosafety protection measures of clinical microbiology laboratory have good prevention and control effect on preventing infection of medical staff, which will greatly reduce the risk of infection of medical staff, form good working habits, and provide reference for biosafety protection of microbiology laboratory during the epidemic of COVID-19.

## 1. Introduction

The complexity of today’s society has brought great difficulties to predict and control the spread of epidemics. It is worth noting that researchers have long recognized that the spread of disease has a network structure: infection spreads from one person to another, taking human contact as the model. The 2019 coronavirus disease (corona virus disease 2019, COVID-19) pandemic is a global public health problem.^[1–7]^ Since December 2019, unexplained pneumonia in Wuhan City, Hubei Province, has been confirmed to be caused by the novel coronavirus (2019-nCoV), the seventh coronavirus that infects humans. On February 11, 2020, the International Committee on Classification of Viruses proposed to name it acute respiratory syndrome coronavirus (SARS-CoV-2),^[8–20]^ which is a kind of coronavirus β. The pneumonia caused by a new member of the genus has been named novel coronavirus pneumonia (NCP) or COVID-19 for short. The WHO has officially named the disease COVID-19. Delta, a variant of the novel coronavirus, has a high infectivity and concealment.^[21,22]^ In the workplace, the physical distance between workers and colleagues and customers is used to measure the risk of exposure to coronavirus. COVID-19 infection spreads through the network with different probabilities among individuals embedded in the society (direct close contact through aerosols or droplets). It is of great significance to understand the role of occupation and workplace environment in the online transmission of COVID-19.

The diagnosis and treatment plan for NCP (trial version 7) takes laboratory examination as one of the important methods for diagnosing NCP. In particular, viral nucleic acid detection is regarded as the “gold standard” for the diagnosis of novel coronavirus infection and is one of the standards for the release of isolation and discharge. Other laboratory examinations are also used as the main basis for observing the disease severity and efficacy. The medical staff of clinical microbiology laboratories is at risk of contact transmission and aerosol transmission during the process of testing patient samples.^[23–33]^ Therefore, personal safety of medical personnel is important for prevention and control.^[34–45]^ According to the relevant documents, guidelines and other materials and the working experience of the designated hospital, and in combination with the current situation of the existing sites, equipment, facilities and personnel of the Department, the corresponding prevention, and control plan is formulated.^[46,47]^ From the perspective of the laboratory, this paper discusses how to do a good job in biosafety protection, improve biosafety protection measures, and optimize the workflow. The purpose of this study was to share the prevention and control measures and processes of clinical microbiology laboratories and provide a reference for inspection peers.

## 2. Methods

### 2.1. Personal protection

#### 2.1..1. *The staff shall be protected by 3-level biosafety protection*.

Wear medical protective masks, disposable hats, protective clothing, protective face screens, double-layer latex gloves, waterproof isolation clothes, and shoe covers.^[48–53]^ See Table 1.

**Table 1:** Wearing and taking off process of personal protective equipment.

Category	Routine
Process of wearing protective articles	1. Hand disinfection
	2. Wear one’s hat
	3. Wear medical protective mask
	4. Wear protective clothing
	5. Protective screen
	6. Wear double latex gloves
	7. Waterproof isolation clothing
	8. Shoe cover
Process of removing protective articles	1. Hand disinfection
	2. Take off gloves
	3. Hand disinfection
	4. Take off the protective screen
	5. Hand disinfection
	6. Take off protective clothing
	7. Hand disinfection
	8. Take off masks and hats
	9. Hand disinfection

#### 2.1..2. *Hand hygiene*.

Master of the correct hand washing method (7-step washing technique) and timing.^[54–57]^ Step 1 (inside): wash your hands, wet your hands with running water, apply hand sanitizer, keep your palms opposite, and rub your fingers together; Step 2 (outside): wash the back finger seam, rub the palm of your hand and the back of your hand along the finger seam, and exchange your hands; Step 3 (clip): wash the palm side finger seam, the palms are opposite, and the hands cross and rub each other along the finger seam; The fourth step is to bend the fingers on the back and rub the fingers on the other hand; Step 5 (big): wash the thumb, hold the thumb of the other hand in one hand, rotate and rub it, and exchange hands; Step 6 (standing): wash the fingertips, bend the finger joints, close the fingertips to the palm of the other hand, rotate and rub, and exchange hands; Step 7 (wrists): wash wrists and arms, rub wrists and arms, and exchange hands. The rubbing time for each step of the hand washing should be greater than 15 seconds.

Recall 5 important aspects of hand hygiene. Two fronts: before contacting the patient and before cleaning/aseptic operation. After contacting the patient, the patient’s body fluid, blood, and secretions, and the patient’s surrounding environment.

### 2.2. *Before inspection*

#### 2.2..1. *Specimen collection*.

All samples were collected in the isolation ward; sputum, urine, and feces were collected by the patients themselves, and other samples such as blood, alveolar lavage fluid, and catheter were collected by doctors or nurses.

#### 2.2..2. *Specimen transportation*.

The specimens were placed into the specimen bag individually by the nurses in the ward, and the surface of the specimen bag was evenly sprayed with 1000 mg/L chlorine-containing disinfectant, transferred to the specimen delivery box, covered, and sealed. The surface of the box was evenly sprayed with 1000 mg/L chlorine-containing disinfectant. After a handover with delivery personnel, it is sent to the delivery window of the inspection department through the pollution channel. They were then sent to the transfer window of the microbiology room by laboratory staff. The transfer box is not opened by yourself during the transfer and keeps the transfer box stable to avoid violent shocks and turbulence.

#### 2.2..3. *Specimen receipt*.

The staff opened the transfer box in a level II biosafety cabinet and sprayed 75% ethanol for disinfection during unpacking. The samples were disinfected before packing. The specimen was removed from the sealed bag and 75% ethanol was sprayed on the surface of the container for disinfection. Signed in for each sample through LIS system registration.

### 2.3. *Under inspection*

#### 2.3.1. *Smears can easily produce droplets and aerosols*.

Communicate with the clinic and reduce detection as much as possible. If necessary, first conduct high-pressure sterilization, then smear, naturally dry, fix, dye, and perform microscopic examination in a biosafety cabinet or smear, UV irradiation for 30 minutes.

#### 2.3.2. *Open the bag and cover in the biosafety cabinet and select the appropriate plate for inoculation*.

The inoculated plate was placed in a sealable specimen bag, and 75% ethanol was sprayed onto the surface for disinfection and incubated in an incubator at 35 to 37 °C. The surface of the blood culture bottle was sprayed with 75% ethanol and placed in an automated blood culture instrument.

#### 2.3..1. *Bacterial identification and drug sensitivity*.

The target plate, formic acid, acetonitrile, and matrix solution were applied, wiped with disinfectant wipes outside the point, and identified by mass spectrometry.

#### 2.3..2. *Drug sensitivity*.

Adjust 0.5 Michaelis unit suspension was added, gently and slowly moved, aerosol was avoided as much as possible, allowed to stand for 30 minutes, placed in the corresponding lath, the surface of the test tube was disinfected, wiped with a wet towel, and the machine was used.

#### 2.3..3. *Quality control*.

According to the internal quality control procedure of the microbiological laboratory.

### 2.4. *After inspection*

#### 2.4..1. *Report*.

The laboratory is a paperless office. The system can be consulted after the report has been reviewed.

#### 2.4..2. *Waste*.

The Sharps box was equipped with 2000 mg/L of chlorine-containing disinfectants. The wastes generated in the biosafety cabinet, such as plates, specimens, inoculation rings, and other disposable items, were discarded and soaked in a sharps box. After the work, put the sharps box in the biosafety cabinet into a closed yellow medical waste bag with biological hazard warning signs, wrap it, and tie it tightly. Use 75% ethanol to fully spray wine on the seal and outside of the yellow medical garbage bag removed from the biosafety cabinet. Quickly cover the outside with another layer of yellow medical waste bag and wrap it tightly.

Garbage outside the biosafety cabinet should be fastened with double-layer closed yellow medical garbage bags. The mixture was placed in an autoclave and sterilized at 121 °C for 30 minutes. Each batch was monitored using a chemical indicator card to determine whether it met the sterilization conditions according to the change in color and shape. After high-pressure sterilization, it is placed into a special transfer window for waste, which is collected and treated by hospital logistics.

#### 2.4..3. *Terminal disinfection*.

Disinfection of floors and walls: If there is no obvious contaminant, use 1000 mg/L chlorine-containing disinfectant mop to wipe and disinfect once per shift. After the visible pollutants are fully covered with disposable rags, pour 2000/L chlorine containing disinfectant on the water-absorbing material, and remove after acting for more than 60 minutes.

Disinfection of the object surface: Corrosion-resistant surfaces, such as desktops and 1000 mg/L chlorine-containing disinfectants, are preferred for wiping and disinfection, whereas noncorrosion-resistant surfaces, such as instrument surfaces, sampling guns, and turbidimeters, can be wiped and disinfected with 75% ethanol.

Air disinfection: Before the end of each shift, the polluted area, buffer area, biosafety cabinet, and transmission window were disinfected by ultraviolet rays for 60 minutes each time.

## 3. Discussion

Since the outbreak of the epidemic, the clinical microbiology room has improved biosafety protection measures and optimized the workflow, which is summarized as follows: strengthen the biosafety training of staff, strictly follow the standard operation when wearing and taking off protective clothing, and remind each other. In particular, you gently take your clothes off and roll them up as much as possible. Smears and cultures can easily produce aerosols or splashes, resulting in transmission. The system should be operated in a biosafety cabinet. During the operation, the hand is extended inward as much as possible, and it is safer to be more than 30 cm away from the air outlet. In the case of direct contact with the specimen, the outer gloves were replaced. Fully disinfect the laboratory daily to prevent the virus from spreading through the air.

We have not yet overcome the epidemic, but at least we can imagine an end. During the novel coronavirus outbreak, medical staff faced significant physical and psychological challenges. Laboratory workers should be stricter in their classification of protection levels, use protective equipment correctly and reasonably, and reduce the impact of waste and inadequate protection. Laboratory staff should encourage and supervise each other and perform good jobs with physical and psychological protection. Although the novel coronavirus is highly infectious, which brings high requirements to the biological safety of the laboratory, as long as the laboratory completes the protection work according to the biological safety protection requirements and laboratory personnel strictly implement it, the pollution caused by the novel coronavirus can be prevented and controlled and strict implementation by laboratory personnel can prevent and control the pollution caused by the novel coronavirus.

## Author contributions

**Investigation:** Fenyong Zhu, Limin Zou.

**Writing—original draft:** Wenjun Zhou.

**Writing—review and editing:** Jie Yang.
